# Plasma miRNAs as Diagnostic and Prognostic Biomarkers for Ovarian Cancer

**DOI:** 10.1371/journal.pone.0077853

**Published:** 2013-11-01

**Authors:** Hong Zheng, Lina Zhang, Yanrui Zhao, Da Yang, Fengju Song, Yang Wen, Quan Hao, Zhibin Hu, Wei Zhang, Kexin Chen

**Affiliations:** 1 Department of Epidemiology and Biostatistics, Tianjin Medical University Cancer Institute and Hospital, Tianjin, P.R. China; 2 Key Laboratory of Breast Cancer Prevention and Therapy, Tianjin Medical University, Ministry of Education, Tianjin, P.R. China; 3 Department of Breast Oncology, Tianjin Medical University Cancer Institute and Hospital, Tianjin, P.R. China; 4 Department of Pathology, The University of Texas MD Anderson Cancer Center, Houston, Texas, United States of America; 5 Key Laboratory of Reproductive Medicine, Department of Epidemiology and Biostatistics, School of Public Health, Nanjing Medical University, Nanjing, China; 6 Department of Gynaecological Oncology, Tianjin Medical University Cancer Institute and Hospital, Tianjin, P.R. China; Deutsches Krebsforschungszentrum, Germany

## Abstract

**Background:**

Most (70%) epithelial ovarian cancers (EOCs) are diagnosed late. Non-invasive biomarkers that facilitate disease detection and predict outcome are needed. The microRNAs (miRNAs) represent a new class of biomarkers. This study was to identify and validate plasma miRNAs as biomarkers in EOC.

**Methodology/Principal Findings:**

We evaluated plasma samples of 360 EOC patients and 200 healthy controls from two institutions. All samples were grouped into screening, training and validation sets. We scanned the circulating plasma miRNAs by TaqMan low-density array in the screening set and identified/validated miRNA markers by real-time polymerase chain reaction assay in the training set. Receiver operating characteristic and logistic regression analyses established the diagnostic miRNA panel, which were confirmed in the validation sets. We found higher plasma miR-205 and lower let-7f expression in cases than in controls. MiR-205 and let-7f together provided high diagnostic accuracy for EOC, especially in patients with stage I disease. The combination of these two miRNAs and carbohydrate antigen-125 (CA-125) further improved the accuracy of detection. MiR-483-5p expression was elevated in stages III and IV compared with in stages I and II, which was consistent with its expression pattern in tumor tissues. Furthermore, lower levels of let-7f were predictive of poor prognosis in EOC patients.

**Conclusions/Significance:**

Our findings indicate that plasma miR-205 and let-7f are biomarkers for ovarian cancer detection that complement CA-125; let-7f may be predictive of ovarian cancer prognosis.

## Introduction

Ovarian cancer is the fifth leading cause of cancer death in women worldwide, and epithelial ovarian cancer (EOC) is the most lethal gynecologic neoplasm [1]. Because EOC is usually asymptomatic and few screening tests are available, almost 70% of women present with advanced-stage (stage III or IV) disease, with 5-year survival rates of less than 30% [2]. In contrast, patients who are diagnosed with stage I disease have a 5-year survival rate of up to 90%, and patients with stage II disease have a 5-year survival rate of up to 70% [3]. Therefore, the early detection of EOC may greatly improve clinical outcomes. Sensitive, non-invasive biomarkers that can facilitate disease detection and staging and predict therapeutic outcome are needed to improve survival rates and determine optimal treatments.

Carbohydrate antigen-125 (CA-125) is the most frequently used biomarker for ovarian cancer detection [4], but it is only elevated in approximately 50% of stage I EOCs and 70%–90% of advanced cases [5]. Hence, there is a critical need for novel biomarkers that are more sensitive and specific for detecting EOC when used alone or in combination with CA-125. Recently, microRNAs (miRNAs), a family of small regulatory RNAs, emerged as possible plasma markers for human disease, including cancer, because of their relative stability in the circulation [6].

MiRNAs are small, non-protein-encoding RNAs that post-transcriptionally regulate gene expression by suppressing specific target mRNAs [7], [8]. Specific circulating miRNAs have been associated with different tumor types, including lung cancer [9], [10], colon cancer [11], [12], prostate cancer [13], ovarian cancer [14], [15], and pancreatic cancer [16], [17]. Taylor *et al.* [14] compared the miRNA profiles of tumor-derived exosomes that had been isolated from the serum of ovarian cancer patients with those from matched tumor tissues from the same patients. However, these studies were limited by having small sample sizes and evaluating few clinical factors. The purpose of this study was to identify and validate circulating miRNAs in human plasma for use as diagnostic and prognostic biomarkers in ovarian cancer. We sought to compare these identifid plasma miRNAs with CA-125, especially to increase the sensitivity of EOC detection in patients with normal CA-125 serum levels.

## Materials and Methods

### Study Design and Population

**Figure 1:**
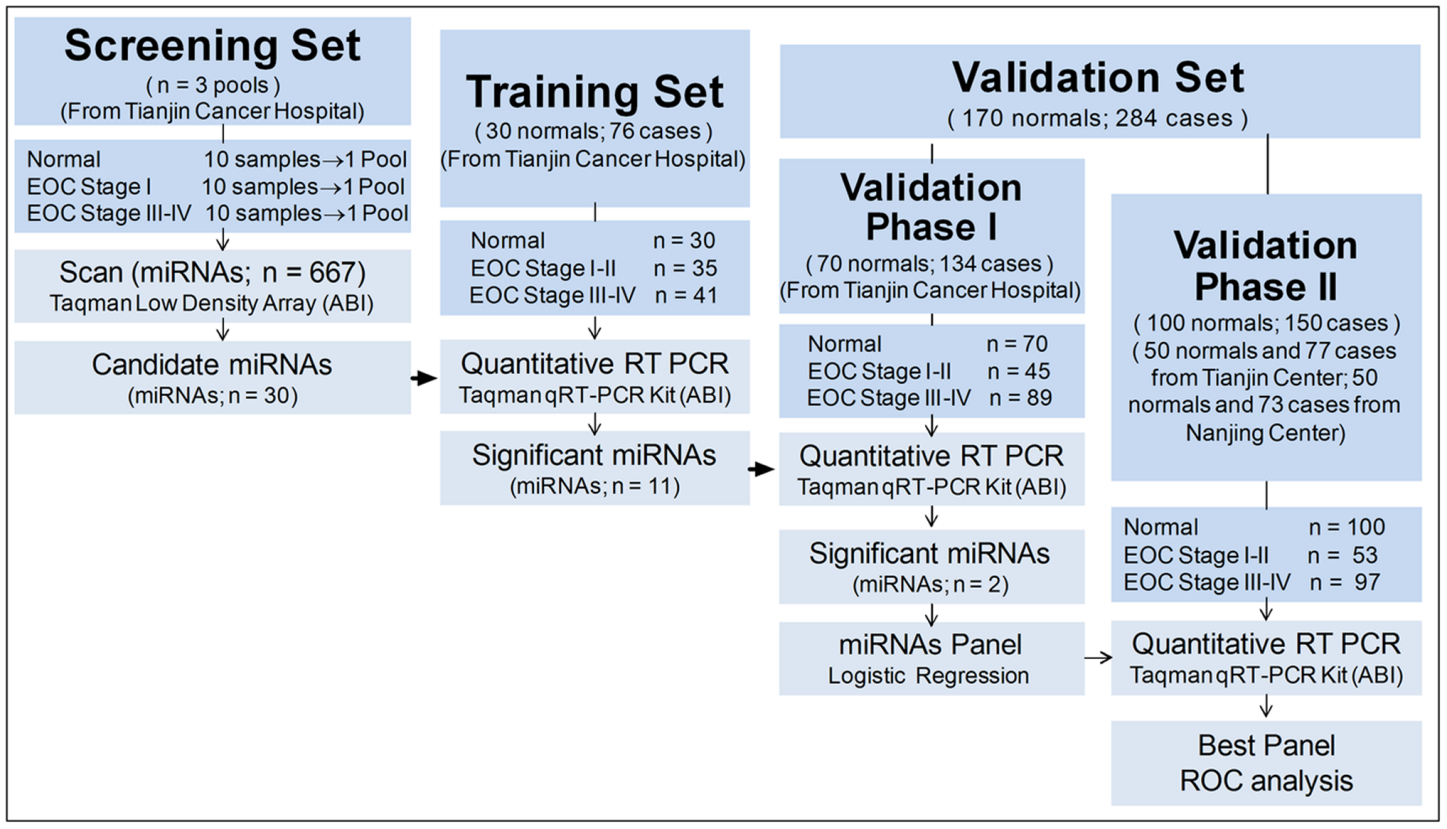
Study design flowchart. We separated the samples into three sets: screening, training, and validation. The validation set included two phases to validate the best panel.

We designed a multi-stage, retrospective, nested case-control study to determine whether serum miRNA profiles can be used to predict EOC development. All samples were grouped into screening, training, and validation sets (Fig. 1). A total of 560 plasma samples (360 cases and 200 controls) were obtained from Tianjin Medical University Cancer Institute and Hospital (TCIH) and Cancer Center of Nanjing Medical University between July 2007 and June 2010. The cases and controls were well matched for age among the training and validation sets. All EOC patients had been histopathologically diagnosed with primary ovarian cancer. All patients recruited to this study had not received chemotherapy or radiotherapy prior to the blood draws. And all blood samples were obtained before surgery. The International Federation of Gynaecology and Obstetrics (FIGO) staging system was used to stage cases. All patients and controls were genetically unrelated, ethnic Han Chinese women who were permanent residents of the urban area of Tianjin and Nanjing. Controls with cardiovascular, respiratory, digestive, urinary, reproductive, and endocrine diseases were excluded.

The study protocols were approved by the Tianjin and Nanjing Center review committees. Informed consent was obtained, and all participants were personally interviewed by trained interviewers using a pre-tested questionnaire to obtain information on demographic data, menstrual and reproductive history, lifestyle, environmental exposure, and family history of cancer. After the interview, a 5-ml venous blood sample was collected. Among these cases, 44 matching ovarian tumor tissue samples were obtained from Tianjin center for this study. EOC tumor tissue samples and tissue RNA were obtained from the Tissue Bank Facility of the TCIH, which collects all solid tumor tissues from surgical patients when possible.

CA-125 levels of plasma samples were acquired from electronic medical records of the two centers. Patients’ follow-up data were acquired, and survival duration was calculated from the date of diagnosis to the date of death or last follow-up in March 2012.

### Screening Set

To detect generalizable miRNA signatures, we pooled serum samples of 10 early-stage cases (stage I), 10 late-stage cases (stage IIIc-IV), and 10 healthy controls, respectively, and analyzed these three pool samples using a TaqMan low-density array (TLDA set 2.0, Applied Biosystems) chip screening in the discovery stage. The TLDA version 2.0 (Sanger miRBase v14 human) included 667 human miRNAs. Microarray data have been deposited in National Center for Biotechnology Information’s (NCBI’s) Gene Expression Omnibus (NCBIGEO GSE50472).

### Training Set

The miRNAs identified in the screening set were validated using quantitative reverse transcription-polymerase chain reaction (qRT-PCR) on individual plasma samples of 76 EOC patients and 30 normal controls.

### Validation Set

The validation set comprised a two-phase process. Promising associations from the screening and training set were evaluated in the validation phase I set, comprising 134 cases and 70 controls from Tianjin Center. A receiver operating characteristic (ROC) curve-based risk assessment analysis was then performed to determine the discriminating effect of candidate plasma miRNAs. The validation phase II set included samples of 77 EOC cases and 50 normal controls were from Tianjin Center and 73 EOC cases and 50 normal controls were from Nanjing Center.

### RNA Isolation

Blood was collected in EDTA tubes (BD Biosciences) and processed to isolate plasma. Blood was centrifuged at 1,200 g at ambient temperature for 10 minutes, and supernatant plasma was transferred to a fresh tube and stored at −80°C.

RNA was isolated from the plasma samples of healthy donors and ovarian cancer patients. In brief, 250 µl of plasma was thawed on ice and spun at 14,000 g at 4°C for 10 minutes to remove lymphocytes. We then lysed 200 µl of supernatant with an equal amount of QIAzol lysis reagent (Qiagen, Shanghai, China). For plasma pools used to screen human ovarian cancer biomarker candidates, we created the pool sample by combining 110 µl of each of 10 samples. The pool was mixed by inversion and spun at 14,000 g for 10 min; 1 ml of supernatant was transferred to a new tube for RNA isolation. For normalization, 5 µl of 25 fmol synthetic *Caenorhabditis elegans* miRNA cel-miR-39 was added to each denatured sample. Small RNA was isolated using miRNeasy Mini (Qiagen) following the manufacturer’s protocol except that RNA was eluted in 30 µl of pre-heated nuclease-free water. To isolate total RNA from ovarian tumor tissues, the frozen samples were first homogenized in TRIzol reagent (Invitrogen, San Diego, CA) using an Omni-Mixer Homogenizer (Omni International, Kennesaw, GA). Total RNA was isolated using the standard TRIZOL method (Invitrogen), according to the manufacturer’s instructions. The quality of total RNA was verified with a Bioanalyzer 2100 (Agilent Technologies).

### MiRNA Profiling

MiRNA expression of the pool sample was profiled using TLDA. In brief, the RNA was reverse transcribed using the TaqMan miRNA reverse transcription kit, and the TaqMan miRNA multiplex RT assays, Human Pool Set. An amount of 9.16 µl of plasma RNA solution was transcribed in 15 µl of RT mixture. The tubes were incubated at 16°C for 30 minutes, followed by 42°C for 30 minutes and 85°C for 5 minutes. The TaqMan PreAmp master mix kit (Applied Biosystems) is intended to increase the quantity of cDNA available for analysis before using the TLDA. The 25 µl reaction mixture consisted of 2.5 µl of undiluted cDNA combined with 12.5 µl of TaqMan PreAmp master mix, 2.5 µl of Megaplex PreAmp primers, and 7.5 µl of nuclease-free water. The pre-amplification step was performed according to the manufacturer’s protocol. The product was diluted by adding 75 µl of nuclease-free water, and 9 µl of the diluted mixture was combined with 450 µl of TaqMan Universal PCR master mix and 441 µl of nuclease-free water. After loading 100 µl of each multiplex pool mixture, the array (TaqMan Human MicroRNA Array Set v2.0) was centrifuged and sealed. Amplification was performed on an Applied Biosystems 7900 HT thermal cycler (Applied Biosystems) using the manufacturer’s recommended cycling conditions. Data were analyzed using RQ Manager Software version 1.2 and 7900 Sequence Detection System 2.4 (Applied Biosystems).

### MiRNA Quantification by Real-time qRT-PCR

To determine miRNA levels in plasma samples, we used the ABI miRT-PCR system, with the following conditions: 2.5 µl of enriched small RNAs from plasma samples or 10 ng of total RNA from tissue samples were reverse transcribed using the TaqMan miRNA reverse transcription kit (Applied Biosystems) in 7.5 µl of reaction buffer with the target-specific primers. The tubes were incubated at 16°C for 30 minutes, followed by 42°C for 30 minutes and 85°C for 5 minutes. After priming, a 1∶20 dilution was used as the template for the PCR stage using TaqMan 2× Universal PCR master mix (Applied Biosystems) on an ABI Prism 7900. We measured triplicate wells using a 15-µl final reaction volume and the following cycling conditions: 10 minutes at 95°C, followed by 50 cycles of 95°C for 15 seconds and 60°C for 1 minute. The 7900 Sequence Detection System 2.4 software defaults were used to compute the relative change in expression by the 2^−ΔΔCt^ method with 95% confidence.

### Statistical Analysis

The relative levels of miRNA were quantified using the 2^−ΔΔCt^ method, and the data were analyzed as the log_10_ of the relative quantity of the target miRNA. The statistical significance was determined using the Wilcoxon signed-rank or *t*-test between patients and controls. ROC curves were generated to assess the diagnostic accuracy of each candidate miRNA, and the sensitivity and specificity of the optimum cut-off point were defined as those values that maximized the area under the ROC curve (AUC). A stepwise logistic regression model was used to select diagnostic miRNA markers on the basis of the training data set. The correlation between survival (overall survival [OS] and progression free survival [PFS]) and plasma miRNAs was analyzed using the Kaplan-Meier method and log-rank test. A Cox proportional hazards regression analysis was used to determine whether plasma miRNA was an independent prognostic factor for EOC.

We used the statistical software packages SAS version 9.0 (SAS Institute, Cary, NC) and MedCalc (Mariakerke, Belgium). The graphs were generated with GraphPad Prism 5.0 (GraphPad Software, Inc., La Jolla, CA). All statistical tests were two-sided, and a *P* value of <0.05 was considered significant.

## Results

### Patient Characteristics

We collected 560 plasma samples (360 cases and 200 controls) and associated clinical and demographic data for this study; the cases included 179 serous tumors (49.7%), 86 endometrioid tumors (23.9%), 33 mucinous tumors (9.2%), 15 clear cell tumors (4.2%), and 47 adenocarcinomas, Not Otherwise Specified (NOS) (13.0%). The participants’ characteristics are presented in Table 1 and Table S1. The distributions of most risk factors were generally similar between patients and controls (Table S1). In patients, there was no significant difference in the distribution of FIGO stages, histologic grade, or CA-125 levels between the training and validation groups (Table 1).

**Table 1:** Pathological and demographic characteristics of EOC patients in the training and validation sets.

Characteristic	Training	Validation I	Validation II	*P*
	(n = 76)	(n = 134)	(n = 150)	
Mean age, years (SD)	56.51 (11.57)	53.75 (10.25)	53.22 (10.38)	0.231
Histologic type, n (%)				
Mucinous	9 (11.8)	14 (10.4)	10 (6.7)	
Serous	37 (48.7)	69 (51.5)	73 (48.7)	
Endometrioid	23 (30.3)	33 (24.6)	30 (20.0)	
Clear cell	4 (5.3)	6 (4.5)	5 (3.3)	
Adenocarcinoma, NOS	3 (3.9)	12 (9.0)	32 (21.3)*	<0.01
FIGO stage, n (%)				
I	23 (30.3)	22 (16.4)	20 (13.3)	
II	12 (15.8)	23 (17.2)	33 (22.0)	
III	36 (47.4)	73 (54.5)	81 (54.0)	
IV	5 (6.6)	16 (11.9)	16 (10.7)	0.071
Histologic grade, n (%)				
I	7 (9.2)	4 (4.8)	2 (4.0)	
II	24 (31.6)	28 (33.7)	12 (24.0)	
III	45 (59.2)	51 (61.5)	36 (72.0)	0.373
Surgery, n (%)				
None	0	0	2 (1.3)	
Salpingo-oophorectomy	13 (17.1)	20 (14.9)	3 (2.0)	
De-bulking surgery (residual disease ≤1 cm)	49 (64.5)	89 (66.4)	56 (37.3)	
De-bulking surgery (residual disease >1 cm)	14 (18.4)	25 (18.7)	16 (10.7)	
Unknown	0	0	71 (47.3)*	–
Chemotherapy, n (%)				
No	10 (13.2)	18 (13.4)	5 (3.33)	
Yes	65 (86.7)	116 (86.6)	111 (74.0)	
Unknown	0	0	34 (22.7)*	–
CA-125 (U/ml), n (%)				
≤35	8 (10.8)	14 (10.7)	14 (12.2)	
>35	66 (89.2)	117 (89.3)	101 (87.8)	0.978

* In phase II, some information was missing for Nanjing Center patients.

### MiRNA Screening

**Figure 2:**
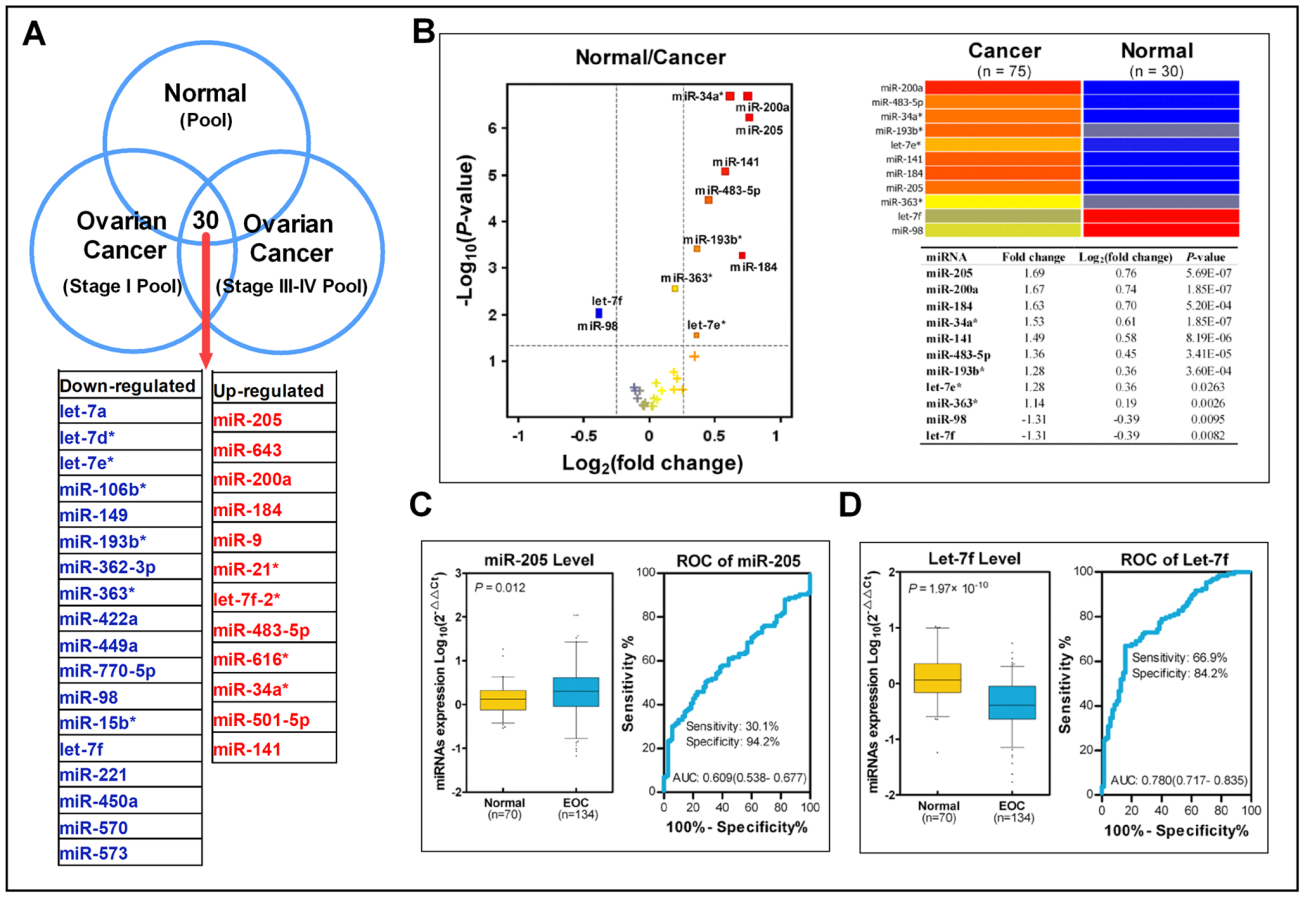
Plasma miRNA expression in cases and controls. (A) A TLDA screening revealed 30 significant plasma miRNA changes between cases and controls. (B) In the training set, we detected 30 significant miRNAs using qRT-PCR and found that 11 candidate miRNAs were differentially expressed by the volcano plot. The -log_10_ of *P* values (y axis) is plotted against the log_2_ of fold change between cases and controls (x axis). Each symbol is color coded according to the mean expression of the probe across the two groups. The dotted lines delineate the cut-offs for significantly downregulated (left) or upregulated (right) miRNAs in cases. A heat map of the 11 significant miRNAs is shown on the right. (C,D) In validation phase I, only miR-205 (C, left) and let-7f (D, left) had differing levels. A ROC analysis indicated that the AUCs of miR-205 and let-7f for differentiating cases and controls were 0.609 (95% CI: 0.538–0.677) and 0.780 (95% CI: 0.717–0.835), respectively.

Using the TLDA chip, we screened 667 human miRNAs to find the significantly different miRNA expression levels between cases and controls. To efficiently screen multiple miRNA biomarker candidates, we first generated three pools of plasma aliquots that had been derived from early- and late-stage EOC patients and healthy controls. Compared with the control pool, there were 43 and 50 miRNAs that were differentially expressed in the early-stage and late-stage case pool, respectively. Among these significant miRNAs, there were 30 miRNAs crossed. Thirty differentially expressed plasma miRNAs were identified between EOC patients and healthy controls (Fig. 2A and Table S2).

### Plasma miRNAs Levels of Patients versus Controls in the Training Set

In the training set, we used qRT-PCR to detect the aforementioned 30 significant miRNAs in a case-control study that included 76 EOC cases and 30 healthy controls. Nine miRNAs (miR-205, miR-200a, miR-184, miR-34a*, miR-141, miR-483-5p, miR-193b*, let-7e*, and miR-363*) were detected at higher levels in cases than in controls, whereas miR-98 and let-7f were detected at lower levels (illustrated by a volcano plot and heat map, Fig. 2B).

### Plasma miRNAs Levels of Patients versus Controls in the Validation Phase I Set

In the validation phase I set, the expression profiles of 11 candidate miRNAs was further evaluated using the qRT-PCR method in 134 EOC cases and 70 controls. MiR-205 and let-7f had different expression levels between cases and controls. Compared with in controls, plasma miR-205 levels were significantly higher (*P* = 0.012) (Fig. 2C, left panel) and let-7f levels were significantly lower (*P* = 1.97×10^−10^) in EOC patients (Fig. 2D, left panel).

A ROC analysis was used to evaluate the sensitivity and specificity of the two candidate plasma miRNA markers. The AUC of miR-205 and let-7f were 0.609 (95% CI, 0.538 to 0.677; sensitivity = 30.1%, specificity = 94.2%) (Fig. 2C, right panel) and 0.780 (95% CI, 0.717 to 0.835; sensitivity = 66.9%, specificity = 84.2%), respectively (Fig. 2D, right panel).

### Combination of miR-205 and Let-7f for EOC Diagnosis

**Figure 3:**
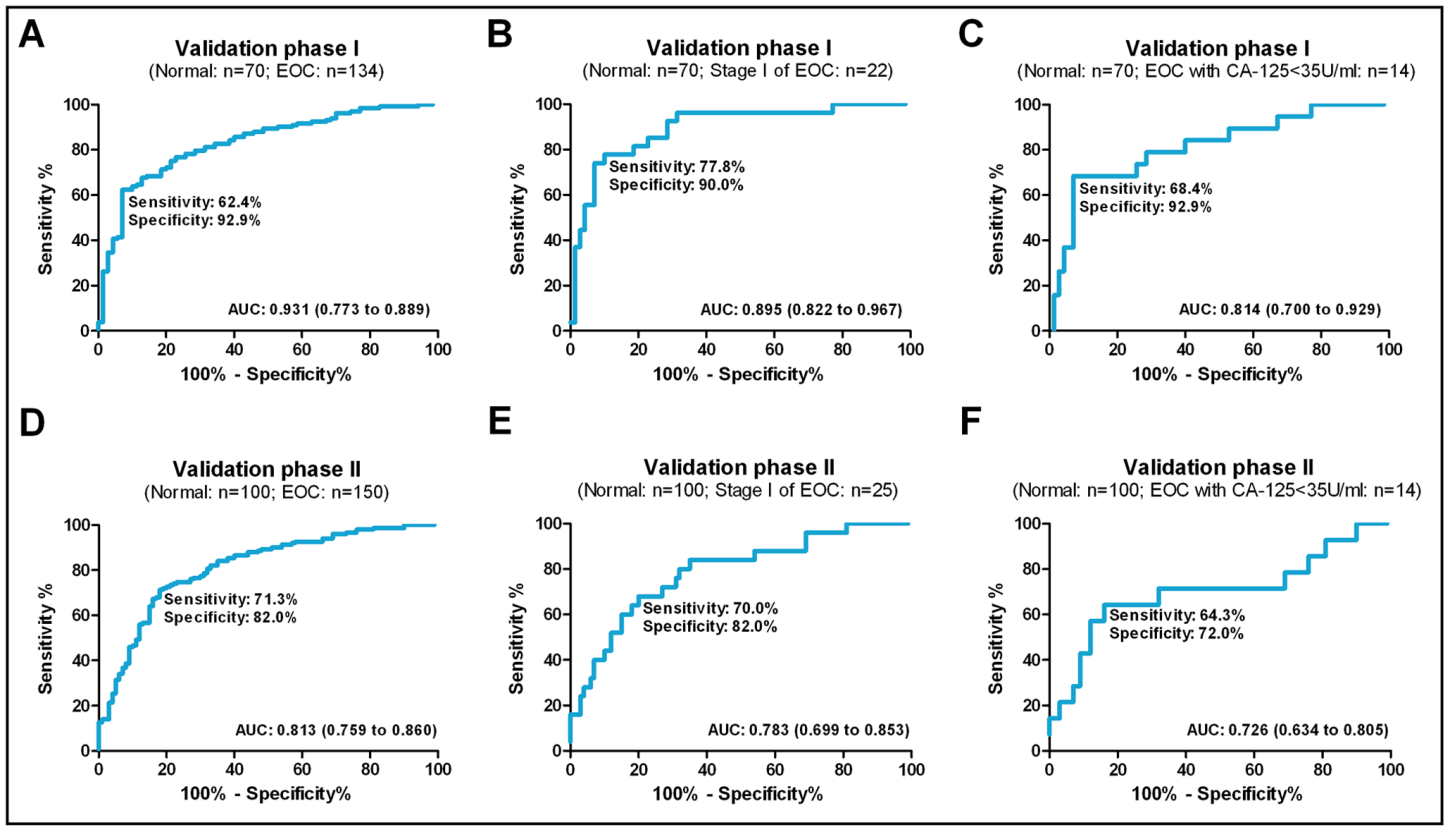
ROC of the panel (miR-205 and let-7f) for EOC cases. We established the miR-205 and let-7f panel in validation phase I and confirmed it in independent samples in phase II. In phase I, (A) the AUC was 0.831 (95% CI, 0.772 to 0.880; sensitivity = 62.4%, specificity = 92.9%) for cases; (B) 0.895 (95% CI, 0.822 to 0.967; sensitivity = 77.8%, specificity = 90.0%) for early-stage cases versus controls; and (C) 0.814 (95% CI, 0.700 to 0.929; sensitivity = 68.4%, specificity = 92.9%) for low CA-125 (<35 U/ml) cases versus controls. In phase II, (D) the AUC was 0.813 (95% CI, 0.759 to 0.860; sensitivity = 71.3%, specificity = 82.0%) for cases versus controls; (E) 0.783 (95% CI, 0.699 to 0.853; sensitivity = 70.0%, specificity = 82.0%) for early-stage cases versus controls; and (F) 0.726 (95% CI, 0.634 to 0.805; sensitivity = 64.3%, specificity = 72.0%) for low CA-125 (<35 U/ml) cases versus controls.

A stepwise logistic regression model was used to estimate the joint power of miR-205 and let-7f at predicting the risk of EOC in the validation phase I set. MiR-205 and let-7f were effective at discriminating cases from controls [Fig. S1A; logit (P = EOC) = −0.610−3.075×let-7f+1.532×miR-205 was used to construct the ROC curve]. The AUC of the miRNA panel (let-7f and miR-205) was 0.831 (95% CI, 0.772 to 0.880; sensitivity = 62.4%, specificity = 92.9%) (Fig. 3A), which was significantly higher than the AUCs of miR-205 (*P*<0.001) and let-7f (*P* = 0.008) alone (Fig. S1B).

The two-miRNA panel also distinguished between early-stage cases (stage I) and controls, with an AUC of 0.895 (95% CI, 0.822 to 0.967; sensitivity = 77.8%, specificity = 90.0%, Fig. 3B). In the validation phase I set, about 89.3% of EOC patients had CA-125 elevation, the other patients failed to be detected by CA-125. The diagnostic accuracy of the miRNA panel was evaluated according to the CA-125 level. In the low CA-125 (<35 U/ml) group (n = 14, 10.7% of all cases, Table 1) in the validation phase I set, the AUC of the miRNA panel was 0.814 (95% CI, 0.700 to 0.929; sensitivity = 68.4%, specificity = 92.9%, Fig. 3C).

### Validating the miRNA Panel in the Validation Phase II Set

The two-miRNA panel was further evaluated in the validation phase II set of 250 plasma samples (150 EOC patients and 100 healthy controls). The AUC of the miRNA panel was 0.813 (95% CI, 0.759 to 0.860; sensitivity = 71.3%, specificity = 82.0%, Fig. 3D) in all validation phase II samples. The AUC was 0.783 (95% CI, 0.699 to 0.853; sensitivity = 70.0%, specificity = 82.0%, Fig. 3E) in early-stage cases (stage I) and controls, respectively. The diagnostic accuracy of the miRNA panel was then evaluated according to the CA-125 level. In the low CA-125 (<35 U/ml) group (n = 14, 9.3% of cases, Table 1) of validation phase II, the AUC of the miRNA panel was 0.726 (95% CI, 0.634 to 0.805; sensitivity = 64.3%, specificity = 72.0%, Fig. 3F).

### Plasma miRNA Levels and Survival

**Figure 4:**
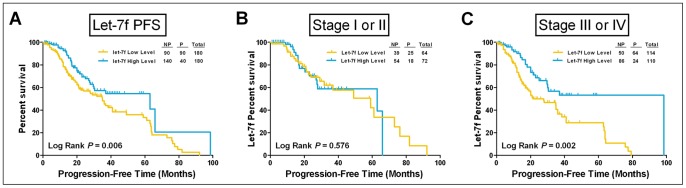
PFS by plasma let-7f level. (A) A PFS analysis for all patients (n = 360) revealed that lower plasma levels of let-7f were associated with poor prognosis (*P* = 0.006). Stage-specific Kaplan-Meier survival curves revealed that the *P* values of the log-rank test were 0.576 and 0.002 for stages I and II (B) and III and IV (C), respectively. The survival data were compared using the log-rank test, and let-7f expression levels in patients were defined as high or low relative to the median. NP, no progression; P, progression.

To further determine whether plasma let-7f and miR-205 levels are predictive of prognosis, we performed an analysis of OS and PFS in all patients from the training and validation sets. A Kaplan–Meier survival curves analysis did not reveal an association between let-7f or miR-205 levels and OS (Fig. S2). However, lower plasma let-7f expression was significantly correlated with poor PFS in all patients (*P* = 0.006, log-rank test) (Fig. 4A), especially in stage III and IV cases (*P* = 0.002, log-rank test). A multivariate Cox regression analysis demonstrated that plasma let-7f was a significant prognostic indicator of EOC in PFS (HR = 2.07, 95% CI = 1.27–3.37) (Table 2).

**Table 2:** Cox regression analysis of EOC OS and PFS in relation to plasma let-7f levels.

Characteristic	PFS				OS			
	Univariate analysis		Multivariate analysis		Univariate analysis		Multivariate analysis	
	HR (95% CI)	*P*	HR (95% CI)	*P*	HR (95% CI)	*P*	HR (95% CI)	*P*
Age	1.02 (1.00–1.04)	**0.040**	1.03 (1.02–1.06)	**0.025**	1.01 (0.99–1.03)	0.142	1.01 (0.98–1.04)	0.482
Stage	1.26 (1.05–1.52)	**0.016**	1.71 (1.28–2.28)	**0.0003**	1.33 (1.09–1.61)	**0.004**	1.96 (1.43–2.68)	**2.82×10^−5^**
Chemotherapy	1.21 (0.71–2.05)	0.491	1.34 (0.76–2.36)	0.308	1.66 (0.89–3.07)	0.109	2.24 (1.12–4.48)	**0.023**
CA-125	1.99 (0.96–4.14)	0.064	1.13 (0.53–2.41)	**0.757**	2.10 (0.97–4.58)	0.062	1.36 (0.61–3.04)	0.460
Let-7f	1.69 (1.26–2.47)	**0.007**	2.07 (1.27–3.37)	**0.003**	1.11 (0.75–1.61)	0.591	1.08 (0.66–1.79)	0.751

### MiRNA Expression in EOC Tissue

We determined whether the expression of miRNAs in plasma at different stages was correlated with that in matching tumor tissues. We examined 11 significant miRNAs (miR-205, miR-200a, miR-184, miR-34a*, miR-141, miR-483-5p, miR-193b*, let-7e*, miR-363*, miR-98 and let-7f) of training set in matched tumor tissue samples. The comparative analysis revealed that the tumor expression of miR-483-5p, but not let-7f, was strongly correlated with the plasma expression (r = 0.541, *P* = 1.52×10^−4^) (Fig. S3A). We also found that miR-483-5p was expressed at higher levels in stage III and IV tumor tissues than in stage I and II (*P* = 0.048) (Fig. S3B), consistent with the levels in plasma (*P* = 0.043) (Fig. S3C). MiR-483-5p was expressed at higher levels in plasma in advanced stages (stage III and IV) than in early stages (stage I and II) (Fig. S3C). No other miRNA levels in plasma were correlated with levels in tumor tissues.

## Discussion

EOC accounts for most ovarian malignancies. Since the discovery of CA-125 in 1981, numerous studies have documented its role as a diagnostic and recurrence tool in EOC patients [18], [19]. Approximately 80% of EOC patients have CA-125 elevation. CA-125 is useful for monitoring disease, assessing therapy response, and detecting relapse [20], [21]. However, it performs poorly in detecting the early stages of ovarian cancer development [22]. New diagnostic markers of ovarian cancer are needed for screening. Currently, several circulating miRNAs have been identified for use in different cancer types [13], [23], [24], [25]. In this study, we found that let-7f and miR-205 are highly promising as diagnostic and progressive biomarkers in EOC.

Using a stringent validation process and a logistic regression model, we demonstrated that the let-7f-miR-205 miRNA panel had high accuracy in EOC diagnosis, especially in patients with stage I disease. Importantly, this panel can be used in low-CA-125 patients to identify early-stage EOC.

The let-7/miR-98 family was one of the first mammalian miRNAs to be identified [26]. Let-7 was originally observed in the nematode *C. elegans* [27], and the expression levels of many let-7-family members have been found to be reduced in various cancers [28], [29], [30], [31], [32]. Let-7f is involved in physiological and pathological processes, including carcinogenesis [33]. Previous studies have demonstrated that it acts as a tumor suppressor in many cancers, including renal cell carcinoma [34], papillary thyroid cancer [35], and gastric cancer [36]. The let-7 miRNA family is selectively secreted into the extracellular environment via exosomes, which may be important in cancer surveillance. A decrease in let-7 in the extracellular environment, such as in plasma, may thus create a cancer- and metastasis-friendly environment. In our study, we found that plasma let-7f can be used as an EOC detection and prognosis biomarker. Compared with healthy controls, ovarian cancer patients have lower levels of plasma let-7f. In EOC patients, these lower levels were also predictive of poor prognosis.

MiR-205 is a highly conserved miRNA among different species, including humans. The miR-200 family (miR-200a, miR-200b, miR-200c, miR-141, and miR-429) and miR-205 are frequently downregulated in advanced cancer [37]. Studies have shown that by targeting the transcriptional repressors of E-cadherin, ZEB1 and ZEB2, the miR-200 family is involved in epithelial-to-mesenchymal transition (EMT) and tumor invasion [38]. Increased expression of miR-205 has also been observed in endometrial adenocarcinoma [39], head and neck squamous cell carcinoma cell lines [40], squamous cell lung carcinoma [41], and ovarian cancer [37]. By contrast, reduced expression of miR-205 has been reported in melanoma [42] and cancers of the esophagus [43], kidney [44], bladder [45], [46], breast [47], and prostate [48]. MiR-205 may function as an oncogene or tumor suppressor gene, depending on cellular context. In our study, plasma miR-205 was expressed at higher levels in EOC patients than in healthy controls. However, there was no difference in miR-205 expression between EOC tumor tissues and adjacent healthy tissues.

The loss of let-7 in cancer results in reverse embryogenesis and dedifferentiation [49], and miR-205 has been identified as a powerful regulator of EMT [50]. Recent findings have connected let-7 with stem cell maintenance and suggest a connection between EMT and stem cell formation. The conversion of an epithelial cell to a mesenchymal cell plays a key role in both embryonic development and cancer invasion and metastasis. Cells undergoing EMT lose their epithelial morphological characteristics, reorganize their cytoskeleton, and acquire a motile phenotype through the up- and down-regulation of several molecules, including tight and adherent junction proteins and mesenchymal markers. In ovarian cancer EMT, let-7f and miR-205 participate in the EMT process by regulating their target genes [49]. Circulating levels of let-7f and miR-205 may be a reflection of the EMT process; thus, it is important to investigate their role in regulating the extracellular environment.

Many studies have demonstrated that circulating miRNAs in blood samples originate from tumor tissues [14], inflammatory foci [51], endothelial cell damage [52], and normal peripheral blood mononuclear cells and platelets [53]. In this study, we analyzed ovarian cancer tissues and found that miR-483-5p expression was correlated with plasma levels. This result suggests that tumor-derived exosomes can explain a small part of the origin of plasma miRNA. In addition, higher miR-483-5p expression was found in advanced EOC than in early EOC, both in tumor tissue and plasma. To our knowledge, this is the first study to find that miR-483-5p may be related to ovarian cancer progression. Recently, serum miR-483-5p was identified as a potential biomarker to detect hepatocellular carcinoma [54].

In summary, we identified plasma let-7f and miR-205 as potential biomarkers for ovarian cancer diagnosis, especially for the early detection of EOC. Let-7f may also be a prognostic marker of ovarian cancer. Circulating miRNAs have been found to be stable, specific, and sensitive biomarkers. On the basis of our data, we propose that specific miRNAs that are associated with circulating exosomes are early markers for ovarian cancer and can be used to determine stage, prognosis, and therapy response.

## Supporting Information

Figure S1 **Diagnostic performance of miRNAs.** (A) The miRNAs were constructed using a logistic regression model. (B) The results of a ROC analysis indicated that the miR-205 and let-7f panel discriminated between cases and controls. The AUC-ROCs of miR-205, let-7, and the miRNA panel (miR-205 and let-7f) for differentiating cases and controls were 0.780, 0.609, and 0.831, respectively. The panel had an AUC = 0.831 (95% CI: 0.772–0.880), which was significantly improved compared with the two miRNAs alone (miR-205 [*P*<0.001] and let-7f [*P* = 0.008]).
(TIF)

Figure S2 **PFS and OS of all patients by let-7f or miR-205 level.** Kaplan-Meier survival curves revealed that low let-7f expression was associated with PFS (*P* = 0.006, Fig. A) but not with OS (*P* = 0.589, Fig. B). There were no differences between miR-205 level and PFS (*P* = 0.969, C) or OS (*P* = 0.576, D). The survival data were compared using the log-rank test, and let-7f or miR-205 expression levels were defined as high or low relative to the median. NP, no progression; P, progression.
(JPG)

Figure S3 **MiRNA expression in EOC tissue.** The expression of miR-483-5p in tumor tissue was highly correlated with the plasma level (r = 0.541, *P* = 1.52×10^−4^) (A). We also found higher miR-483-5p expression in stage III and IV cases than stage I and II cases (*P* = 0.048) (B), consistent with plasma levels (*P* = 0.043) (C).
(JPG)

Table S1 **Characteristics of cases and controls in the training and validation phase I sets.**
(DOC)

Table S2 **Training data (30 miRNAs from screening).**
(DOC)
