# Supplementary figures and images for: Plasma miRNAs as Diagnostic and Prognostic Biomarkers for Ovarian Cancer
Source: PLoS One. 2013 Nov 1;8(11):e77853. doi: 10.1371/journal.pone.0077853 (PMC3815222; doi:10.1371/journal.pone.0077853)

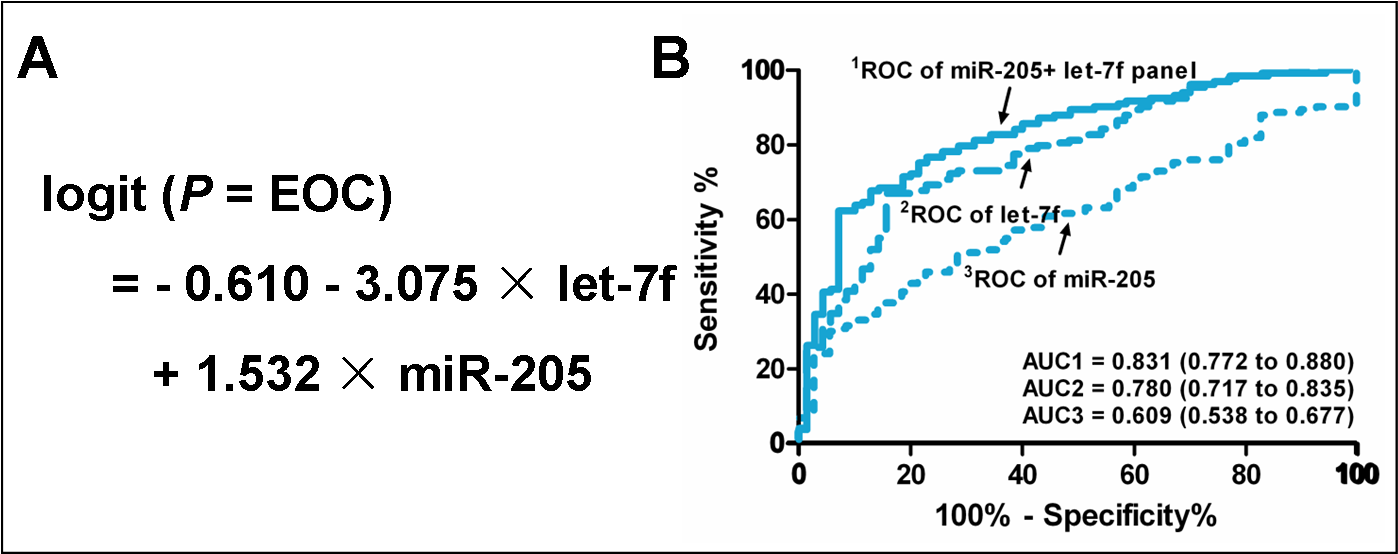

Supplement: Figure S1 — Diagnostic performance of miRNAs. (A) The miRNAs were constructed using a logistic regression model. (B) The results of a ROC analysis indicated that the miR-205 and let-7f panel discriminated between cases and controls. The AUC-ROCs of miR-205, let-7, and the miRNA panel (miR-205 and let-7f) for differentiating cases and controls were 0.780, 0.609, and 0.831, respectively. The panel had an AUC = 0.831 (95% CI: 0.772–0.880), which was significantly improved compared with the two miRNAs alone (miR-205 [P<0.001] and let-7f [P = 0.008]). (TIF) [file pone.0077853.s001.tif]

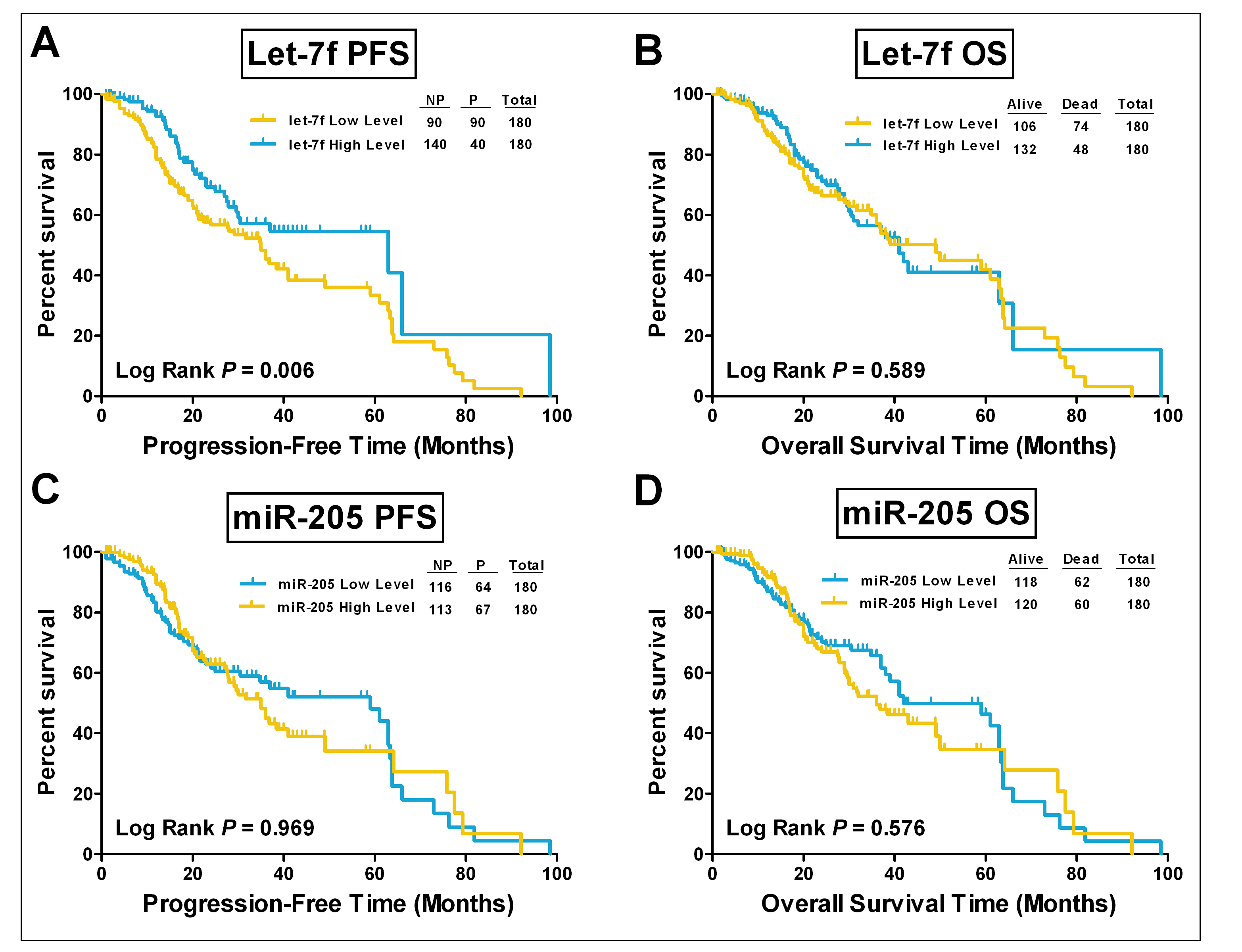

Supplement: Figure S2 — PFS and OS of all patients by let-7f or miR-205 level. Kaplan-Meier survival curves revealed that low let-7f expression was associated with PFS (P = 0.006, Fig. A) but not with OS (P = 0.589, Fig. B). There were no differences between miR-205 level and PFS (P = 0.969, C) or OS (P = 0.576, D). The survival data were compared using the log-rank test, and let-7f or miR-205 expression levels were defined as high or low relative to the median. NP, no progression; P, progression. (JPG) [file pone.0077853.s002.jpg]

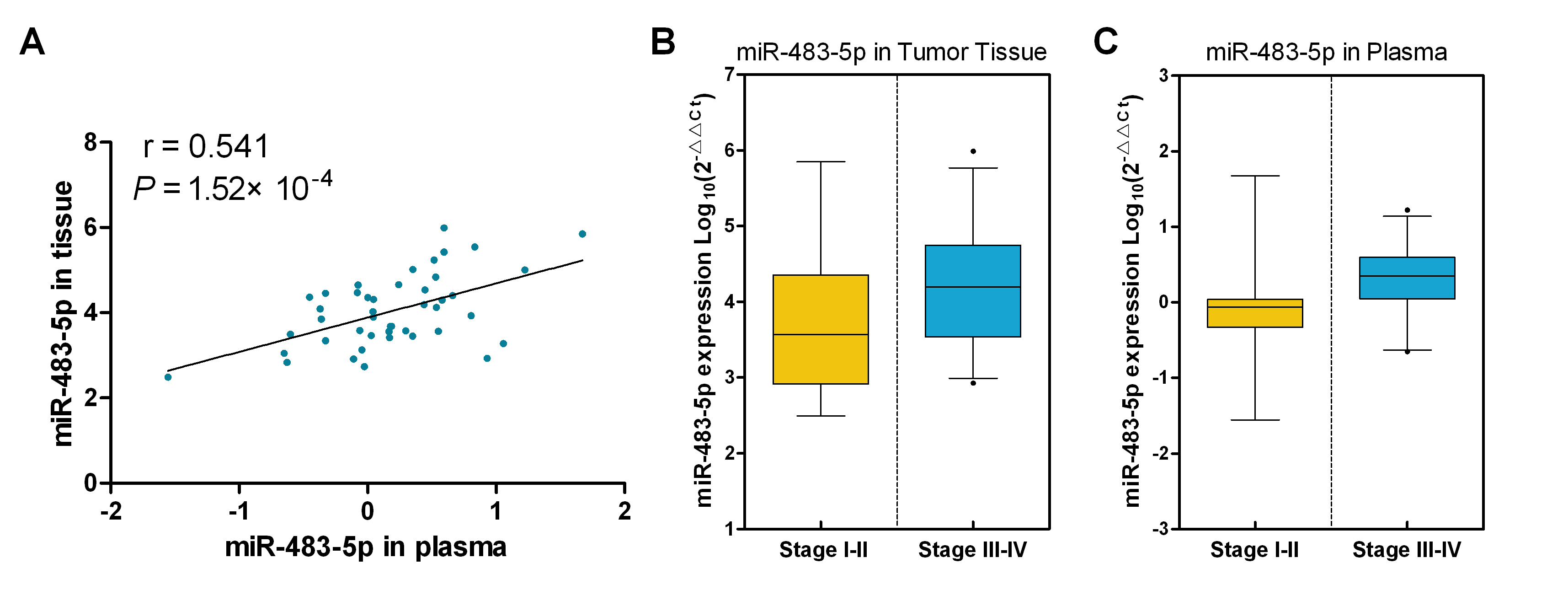

Supplement: Figure S3 — MiRNA expression in EOC tissue. The expression of miR-483-5p in tumor tissue was highly correlated with the plasma level (r = 0.541, P = 1.52×10^−4^) (A). We also found higher miR-483-5p expression in stage III and IV cases than stage I and II cases (P = 0.048) (B), consistent with plasma levels (P = 0.043) (C). (JPG) [file pone.0077853.s003.jpg]
