# Supplementary material for: Plasma miRNAs as Diagnostic and Prognostic Biomarkers for Ovarian Cancer
Source: PLoS One. 2013 Nov 1;8(11):e77853. doi: 10.1371/journal.pone.0077853 (PMC3815222; doi:10.1371/journal.pone.0077853)
Supplement: Table S1 — Characteristics of cases and controls in the training and validation phase I sets. (DOC) [file pone.0077853.s004.doc]

**Table S1.** Characteristics of cases and controls in the training and validation phase I sets

| **Characteristic** | **Training set** | | | **Validation set I** | | |
| --- | --- | --- | --- | --- | --- | --- |
|  | **Control (n = 30)** | **EOC (n = 76)** | ***P*_1_** | **Control (n = 70)** | **EOC (n = 134)** | ***P*_2_** |
| Age (years) at diagnosis^*^ | 54.40 ± 12.58 | 56.51 ± 11.57 | 0.411 | 55.17 ± 11.19 | 53.75 ± 10.25 | 0.365 |
| Body mass index (kg/m^2^) ^*^ | 23.99 ± 3.74 | 24.56 ± 3.75 | 0.489 | 24.69 ± 3.62 | 24.62 ± 3.44 | 0.895 |
| Age (years) at menarche^*^ | 15.50 ± 2.56 | 15.05 ± 2.01 | 0.345 | 15.57 ± 2.46 | 14.71 ± 2.06 | **0.013** |
| Age (years) at menopause^*^ | 47.57 ± 3.93 | 49.95 ± 3.23 | **0.006** | 47.02 ± 5.08 | 48.31 ± 5.84 | 0.210 |
| CA-125 (U/ml) ^*^ | 10.67 ± 5.58 | 749.0 ± 1290.3 | **5.14×10^-6^** | 10.56 ± 4.83 | 1103.8 ± 1634.7 | **3.88×10^-12^** |
| Menopause^†^ |  |  |  |  |  |  |
| No | 7 (23.3) | 18 (24) |  | 20 (28.6) | 51 (41.8) |  |
| Yes | 23 (76.7) | 57 (76.0) | 0.942 | 50 (71.4) | 71 (58.2) | 0.068 |
| Born^†,1^ |  |  |  |  |  |  |
| No | 0 (0) | 3 (4.2) |  | 0 (0) | 2 (1.5) |  |
| Yes | 30 (100) | 69 (4.2) | 0.256 | 70 (100) | 129 (98.5) | 0.302 |
| Family history of cancer^†^ |  |  |  |  |  |  |
| No | 28 (93.3) | 62 (71.3%) |  | 70 (100) | 98 (74.8) |  |
| Yes | 2 (6.7) | 14 (18.4) | 0.128 | 0 (0) | 33 (25.2) | **4.37×10^-6^** |
| CA-125 (U/ml)^†^ |  |  |  |  |  |  |
| ≤ 35 | 30 (100) | 8 (10.8) |  | 70 (100) | 14 (10.7) |  |
| > 35 | 0 (0) | 66 (89.2) | **1.15×10^-7^** | 0 (0) | 117 (89.3) | **2.12×10^-34^** |

^*^ Mean ± SD

^†^ Number (%)

P-value of EOC patients and controls was shown as *P_1_* in training set, as *P_2_* in validation set I.

^1^Born presents previous pregnancies.
