# Supplementary material for: Plasma miRNAs as Diagnostic and Prognostic Biomarkers for Ovarian Cancer
Source: PLoS One. 2013 Nov 1;8(11):e77853. doi: 10.1371/journal.pone.0077853 (PMC3815222; doi:10.1371/journal.pone.0077853)
Supplement: Table S2 — Training data (30 miRNAs from screening). (DOC) [file pone.0077853.s005.doc]

**Table S2.** Training data (30 miRNAs from screening)

| **miRNA** | **Control (n = 30)** | | **EOC (n = 76)** | | ***P*** |
| --- | --- | --- | --- | --- | --- |
|  | **Mean±SD** | **Median** | **Mean****±SD** | **Median** |  |
| let-7a | 10.47±1.60 | 10.46 | 10.34±2.76 | 10.57 | 0.474 |
| let-7d* | 9.91±1.36 | 9.74 | 10.07±1.66 | 9.87 | 0.806 |
| **let-7e*** | 16.37±3.36 | 16.39 | 15.18±2.67 | 15.17 | **0.010** |
| miR-106b* | 13.76±2.00 | 13.41 | 14.06±2.10 | 13.61 | 0.487 |
| miR-149 | 16.97±3.15 | 16.40 | 16.76±1.96 | 16.61 | 0.911 |
| **miR-184** | 20.11±4.65 | 18.10 | 17.78±3.52 | 17.08 | **1.21×10^-4^** |
| **miR-193b*** | 18.11±1.19 | 18.21 | 16.93±1.34 | 16.74 | **7.20×10^-5^** |
| miR-362-3p | 14.24±1.15 | 14.07 | 14.06±2.41 | 13.76 | 0.145 |
| **miR-363*** | 16.75±1.18 | 16.68 | 16.11±2.82 | 15.75 | **0.001** |
| miR-422a | 20.96±2.17 | 20.63 | 20.25±1.42 | 20.23 | 0.113 |
| miR-449a | 18.40±4.50 | 17.28 | 17.56±4.01 | 16.95 | 0.253 |
| miR-501-5p | 13.51±1.26 | 13.34 | 13.40±2.04 | 13.33 | 0.888 |
| miR-770-5p | 18.90±3.21 | 18.65 | 17.94±2.08 | 17.28 | 0.062 |
| **miR-98** | 11.74±1.80 | 11.47 | 13.04±2.12 | 12.68 | **0.003** |
| miR-15b* | 14.47±2.30 | 13.78 | 14.72±2.10 | 14.57 | 0.306 |
| **miR-141** | 18.21±1.83 | 17.84 | 16.30±1.70 | 16.36 | **1.09×10^-6^** |
| **let-7f** | 13.52±1.40 | 13.50 | 14.81±1.94 | 14.58 | **0.002** |
| miR-643 | 17.80±1.15 | 17.63 | 17.47±1.94 | 17.42 | 0.283 |
| miR-9 | 18.54±1.32 | 18.37 | 17.92±1.63 | 18.08 | 0.074 |
| miR-21* | 16.19±1.56 | 16.17 | 16.55±1.57 | 16.42 | 0.290 |
| **miR-34a*** | 19.25±0.70 | 19.34 | 17.22±1.83 | 17.47 | **1.00×10^-8^** |
| **miR-200a** | 18.60±0.97 | 18.63 | 16.13±2.04 | 16.26 | **1.23×10^-8^** |
| **miR-205** | 19.55±1.02 | 19.35 | 17.02±2.47 | 17.15 | **5.68×10^-8^** |
| miR-221 | 18.65±1.77 | 18.50 | 18.02±2.41 | 18.28 | 0.242 |
| miR-450a | 16.21±1.93 | 16.63 | 16.33±1.99 | 16.02 | 0.774 |
| **miR-483-5p** | 16.82±0.85 | 16.94 | 15.33±1.79 | 15.55 | **5.69×10^-6^** |
| miR-570 | 18.06±1.43 | 17.93 | 17.86±1.92 | 17.91 | 0.523 |
| miR-573 | 19.21±1.69 | 19.05 | 19.34±1.70 | 19.05 | 0.855 |
| let-7f-2* | 18.52±1.63 | 18.35 | 18.91±1.65 | 18.85 | 0.202 |
| miR-616* | 16.72±1.01 | 16.54 | 16.83±1.27 | 16.80 | 0.664 |

The yellow miRNAs were up-regulated, and the blue miRNAs were down-regulated.
